# Increased Matrix Metalloproteinase-2 and Matrix Metalloproteinase-3 Concentrations in Corneal Epithelium of Patients with Recurrent Corneal Erosions

**DOI:** 10.1155/2022/5024037

**Published:** 2022-09-25

**Authors:** Katarzyna Jadczyk-Sorek, Wojciech Garczorz, Beata Bubała-Stachowicz, Tomasz Francuz, Ewa Mrukwa-Kominek

**Affiliations:** ^1^Department of Ophthalmology, Gibiński University Clinical Center, Medical University of Silesia, Katowice 40-514, Poland; ^2^Department of Biochemistry, Faculty of Medical Sciences, Katowice, Medical University of Silesia, Katowice 40-027, Poland; ^3^Department of Ophthalmology, Faculty of Medical Sciences, Katowice, Medical University of Silesia, Katowice 40-514, Poland

## Abstract

**Purpose:**

To assess the role of selected matrix metalloproteinases in defective corneal re-epithelization in patients with recurrent corneal erosions.

**Subjects:**

The study group (group 1) included patients with recurrent corneal erosions qualified for phototherapeutic keratectomy. The group 1 was divided into two subgroups regarding the etiology of recurrent corneal erosions: group 1A, Cogan's basement membrane dystrophy, and group 1B, trauma. The control group (group 2) included patients with healthy eyes qualified for Epi-Bowman Keratectomy.

**Methods:**

The analyzed material was the corneal epithelium collected during phototherapeutic keratectomy or Epi-Bowman Keratectomy in the study or control group, respectively. Matrix metalloproteinases concentration was determined by an immunohistochemical method using Human Magnetic Luminex® Assay.

**Results:**

The study revealed a statistically significantly higher concentration of matrix metalloproteinase-2 in group 1 compared to the control and a statistically significantly higher concentration of matrix metalloproteinase-3 in group 1 compared to the control.

**Conclusions:**

The results obtained in the study can prove that matrix metalloproteinase-2 and matrix metalloproteinase-3 having the ability to dissolve anchoring fibers and the corneal epithelial basement membrane could be responsible for epithelial instability and their accumulation in the corneal epithelium may induce recurrence of erosion.

## 1. Introduction

Recurrent corneal erosions (RCEs) are a common disorder characterized by recurrent episodes of spontaneous corneal epithelium breakdown. The syndrome is associated with severe ocular pain (disproportionate to the extent of pathology), photophobia, lacrimation, and decreased visual acuity. RCEs frequently follow some traumatic corneal abrasion with a fingernail, piece of paper, or plant branch. However, they may also occur as a hereditary condition, most commonly epithelial basement membrane dystrophy, also referred to as Cogan's microcystic dystrophy. More rarely, RCEs have been observed in the course of some other dystrophies involving corneal epithelium (Meesmann corneal dystrophy), Bowman's layer (Reis–Bücklers corneal dystrophy, Thiel–Behnke corneal dystrophy), or corneal stroma (lattice, macular, and granular corneal dystrophies), as well as in patients with endothelial dystrophy associated with corneal endothelial decompensation and bullous keratopathy [1, 2]. RCEs can also be diagnosed in patients with severe dry eye syndrome (DES), corneal stromal cell loss, Meibomian gland dysfunction (MGD), and abnormalities of eyelid anatomy [3, 4] or can develop as a complication following surgical correction of refractive errors or corneal transplantation [1]. The clinical course of RCEs can be influenced by systemic disorders including metabolic, hormonal, autoimmune, or neoplastic conditions [1, 5]. An increased predisposition to this disease has been shown in people with rosacea and diabetes [1].

The present day treatment algorithm for RCEs mostly involves medical therapies and, in case of failure, a surgical intervention. First-line therapy is eye lubrication using preservative-free eye drops, gels, or ointments and hypertonic solutions that help reduce corneal edema through osmosis [1]. Local antibiotics and cycloplegics can be used in the acute phase to prevent secondary infection. A bandage contact lens (BCL) may reduce pain, protect the regenerating corneal epithelium against any mechanical damage, and facilitate its adhesion. However, there is no evidence the lens might prevent subsequent recurrence [6–8]. Several authors emphasize the role of autologous serum therapy which seems effective in reducing the number of recurrences. Autologous serum provides fibronectin and thereby promotes epithelial migration and anchorage [9, 10].

Indications for surgery include delayed epithelial healing and failure of medical therapies [11, 12]. Surgical treatment aimed at the removal of nonadhesive corneal epithelium and the promotion of epithelial-stromal attachment. Surgical options include epithelial debridement, superficial keratectomy, diamond burr superficial keratectomy (DBSK) [1, 13], alcohol delamination (ALD) [14, 15], and excimer laser photoablation commonly referred to as phototherapeutic keratectomy (PTK) [5, 12, 16, 17] and currently regarded as the most efficient for RCE treatment.

The latest Cochrane reviews aimed at investigating a wide variety of prophylactic and therapeutic options for RCEs revealed that none of the methods used turned out fully efficient. Hence, there was no evidence for the development of global guidelines for the management of recurrent corneal erosions [2]. Further investigations to establish RCE pathogenesis and treatment options are therefore well warranted.

There are several hypotheses regarding the molecular background of RCEs. It is believed this pathology could be triggered by hemidesmosome deficiency, nonadhesion of corneal epithelium, or structural abnormalities within the cornea [18, 19]. Matrix metalloproteinase (MMP) enzymes might also play a role as they degrade junctional complexes that help the attachment of epithelial cells to the basement membrane [20–22].

Matrix metalloproteinases (MMPs) are proteolytic enzymes that belong to a family of zinc-dependent endopeptidases. The majority are multidomain enzymes containing a catalytic domain, a prodomain, a hemopexin-like domain, and a linker region between the catalytic and hemopexin-like domains. MMPs are secreted as inactive proenzymes; the prodomain retains substrate cleavage in the catalytic pocket due to cysteine interaction with the catalytic zinc ion. MMPs are activated when the inhibitory prodomain is removed and the catalytic site of the enzyme becomes exposed [23, 24]. MMPs are secreted in the connective tissue, leukocytes, macrophages, fibroblasts, vascular endothelium, and neoplastic cells. The activity of MMPs is regulated at various levels. MMP gene expression is controlled by growth factors and cytokines; it can be suppressed by transforming tissue factors (TGF-*β*) and glucocorticoids. Promatrix metalloproteinases can be activated by various proteolytic enzymes (e. g. plasmin, and thrombin) and active MMPs (e.g., MMP-1, MMP-7, and MMP-13). MMP inhibition, on the other hand, occurs via specific tissue inhibitors of metalloproteinases (TIMPs) and nonspecific inhibitors found in human plasma, i.e., *α*2-macroglobulin and *α*1-antiprotease [25–27]. MMPs are capable of degrading the components of the extracellular matrix (ECM) including collagen, fibronectin, and proteoglycans. Thus, physical barriers are disrupted, cell migration accelerates, and bioactive molecules are released from the EMC reservoir. Surface proteins of basement membrane cells are also substrates for MMPs. Hence, the synthesis and release of numerous cytokines, growth factors (e.g., TGF *β*), insulin-like growth factor 1 (IGF-1), hormone receptors, and adhesion molecules are also regulated. MMPs contribute to both physiological (morphogenesis, angiogenesis, and tissue remodeling) and pathological (inflammation, allergy, and fibrosis) processes [23, 25, 26, 28].

RCE development is strongly related to poor epithelium anchorage. We hypothesized this defective adhesion might be induced by anchoring fibril and corneal epithelial basement membrane dissolution by MMPs. Further accumulation thereof can lead to recurrent episodes of the disease. We evaluated differences in the concentrations of selected MMPs in the corneal epithelium of patients diagnosed with RCEs including Cogan's microcystic dystrophy and posttraumatic corneal erosions and those with normal epithelial-stromal complex.

## 2. Materials and Methods

The Medical University of Silesia Research Ethics Committee ruled that approval was not required for this study since the study was designed to use clinical waste.

The analysis comprised 121 eyes of 121 patients allocated to the study or control group. The study group (group 1) comprised PTK candidates with recurrent corneal erosions, subsequently divided into 1A (Cogan's microcystic dystrophy, *n* = 22) and 1B (posttraumatic corneal erosions, *n* = 34). The control group (group 2, *n* = 65) included patients qualified for Epi-Bowman Keratectomy (EBK), i.e., stable epithelial-stromal interface.

### 2.1. Methods of Study Material Collection, Storage, and Sample Preparation

Both PTK and EBK involve the removal of the corneal epithelium, which is then managed according to standard procedures. In our study, the removed epithelium was subjected to biochemical analysis to determine the concentration of selected metalloproteinases.

**Figure 1:**
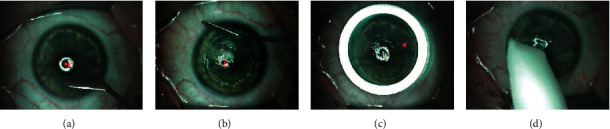
Steps of PTK.

In the study group, during the PTK procedure, following local anesthesia with proxymetacaine solution (0.5% Alcaine®, Alcon), corneal epithelium was harvested over the entire corneal surface with a hockey stick knife (Figure 1(a)) while sparing limbal stem cells. The epithelium was debrided from the periphery to the center (Figure 1(b)) and placed into a color-coded vial property labeled for each patient. Excimer laser photoablation was carried out (Figure 1(c)). Antibiotic eyedrops were instilled and a bandage contact lens was placed (Figure 1(d)).

In the control group, the samples were collected during EBK which was selected to ensure maximum homogeneity of the study material in both groups.

After local anesthesia with proxymetacaine solution (0.5% Alcaine®, Alcon), corneal epithelium was debrided over the entire corneal surface from the periphery to the center using a manual Epi-Clear™ epikeratome with a single-use blade; limbal stem cells were left intact (as in PRK). Epithelial cells collected in the epikeratome were then transferred into vials properly labeled for each eye. Subsequent stages of the procedure were carried out according to the standard protocol. In both PTK and EBK, photoablation was performed with the MEL 80 Excimer Laser (Carl Zeiss-Meditec, Germany).

Epithelial cells debrided in the study and control groups were placed into 1.8 ml Cryovials (Chem-Land, Poland) manufactured of high-quality polypropylene designed for storing biological material and withstanding temperatures as low as −196°C. To protect enzymes contained in the samples against thermal degradation, the vials were put on ice and stored at −80°C until assay.

### 2.2. Evaluation of MMPs Concentrations in the Corneal Epithelium with Human Magnetic Luminex® Assay

Based on the results of the pilot study and substrate specificity profiling, six MMPs were selected for concentration assessments, i. e., MMP-1, MMP-2, MMP-3, MMP-7, MMP-8, MMP-9. Samples were lysed in PathScan lysis buffer (Cell Signaling Technology) containing phenylmethylsulfonyl fluoride (Sigma) and protease inhibitor (Roche).

MMPs concentrations were determined with Human Magnetic Luminex® Performance Assay MMP Kit (R&D Systems, Bio-Techne, USA) according to the manufacturer's instructions. The system allows simultaneous detection and quantification of multiple target analytes in each well of a 96-well microplate. Sample volumes are very small. Human Magnetic Luminex® Performance Assay MMP Kit is the fastest to deliver a precise sample profile while ensuring high accuracy and reproducibility.

In 95–100% of the patients, the concentrations of MMP-1,-7,-8,-9 were below Human Magnetic Luminex® Performance Assay MMP Kit sensitivity limits. The results would not, therefore, be reliable and no further biochemical analyses were carried out. Detailed analyses were conducted regarding the concentrations of MMP-2 and MMP-3.

### 2.3. Statistical Analysis

The values of continuous variables were presented as means with standard deviation and median with quartile range (lower and upper quartile). The minimum and maximum values were also given after removing the extreme values. The Shapiro-Wilk test was used to assess the distribution of continuous variables, while the differences between the two groups were assessed using the U–Mann–Whitney test in the case of the distribution of the variable deviating from the normal and t-student in the case of variables with a distribution close to normal. To evaluate the differences in the range of MMP-2 and MMP-3 between groups 1A, 1B, and 2, the nonparametric ANOVA test was used, the Kruskal–Wallis test. The Bonferroni correction was used for multiple comparisons (post hoc analysis). Then, a two-way analysis of variance and generalized linear models were performed to assess the simultaneous effect of variables such as gender, RCE duration, or age when sampled on MMP-2 and MMP-3 values. The homogeneity of variance was assessed with the Levene test.

The prevalence of features (qualitative variables) was presented as percentage values and important *N* values. To compare the frequency of the trait chi-square test was used in groups/subgroups, Yates's correction was used if necessary, or Fisher's test for small numbers was used. To assess the correlation between continuous variables the R-Spearman rank test was used. Statistical significance was assumed at the level of *P* < 0.05. Statistical analysis was performed with the use of Statistica 13.0 PL (StatSoft Polska, Kraków, Poland) and SAS 9.3 (Institute INC. Cary, USA).

## 3. Results

### 3.1. Patient Characteristics

The study and control groups included 72 women (59.5%) and 49 men (41.5%). The proportion of men was the highest in group 2 (*n* = 25; 51%) followed by 1B (*n* = 17; 34.7%), and the lowest in 1A (*n* = 7; 14.3%). The proportion of women was the highest in group 2 (*n* = 40; 55.6%) followed by 1B (*n* = 17; 23.6%) and 1A (*n* = 15; 20.8%). An analysis revealed that RCE prevalence did not differ significantly between male and female patients of 1A and 1B groups (chi^2^ = 2.07, df = 2, *P*=0.3).

Fingernail injury was the most common cause (41%, *n* = 14) of RCE in the traumatic group; other injuries (59%) were sustained by 20 patients. The prevalence of injury type did not differ between male and female participants (Yates corrected X^2^ (1, *N* = 34) = 0.121, *P*=1).

The mean age of 1A, 1B, and group 2 patients was 54.44 ± 15.0, 36.2 ± 9.7, and 28 ± 5.6 years, respectively. Hence, 1A patients were significantly older than 1B participants; group 2 patients were the youngest (chi^2^ = 36.23, df = 2), *P* < 0.001. Regarding age at RCE onset, the 1B group was slightly younger than 1A; the difference proved statistically significant *U* = (N _1A_ = 22, N _1B_ = 34) = 109; *Z* = 4.43; *P*=0.00009.

An analysis of pre-PTK symptom duration showed that, in 1A patients, the time span between disease onset and PTK referral was by 4.5 months longer compared to 1B participants. However, the difference did not reach the level of statistical significance *U* = (N_1A_ = 22, N_1B_ = 34) = 322; *Z* = 0.86; *P*=0.3.

Epithelial instability at 7 days of PTK was more prevalent in 1A (41%; *n* = 9) compared to 1B (26%; *n* = 9), the difference being statistically nonsignificant X^2^ (1, *N* = 56) = 1.28, *P*=0.2.

The results are presented in Table 1.

#### 3.1.1. MMP-2 oncentrations in the Corneal Epithelium

**Figure 2:**
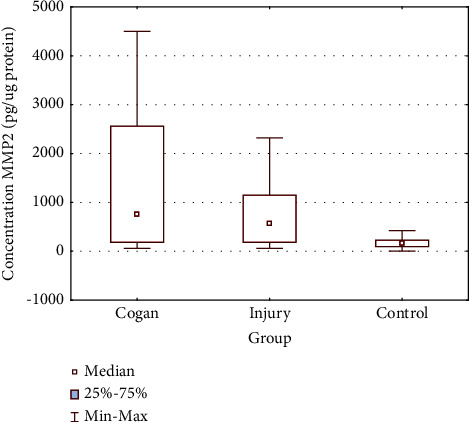
MMP-2 concentration values in the studied groups.

Post hoc analyses revealed a higher concentration of MMP-2 in group 1A compared to the control as well as in group 1B compare to the control. The differences turned out statistically significant KW-H (2, *N* = 106) = 24.47583, *P*=0.0001. The results are presented in Figure 2 and Table 2. No significant differences were found between MMP-2 concentration levels in 1A and 1B groups.

#### 3.1.2. MMP-3 Concentrations in the Corneal Epithelium

**Figure 3:**
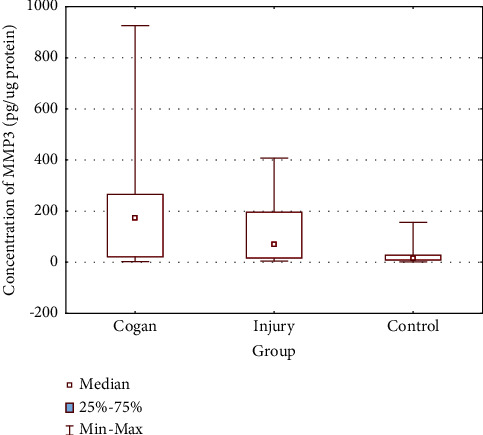
MMP-3 concentration values in the studied groups.

Post hoc analyses revealed higher concentrations of MMP-3 in group 1A compared to the control as well as in group 1B compare to the control. The differences turned out statistically significant KW-H (2, *N* = 90) = 20.79593, *P* < 0.0001. The results are presented in Figure 3 and Table 2. No significant differences were found between MMP-3 concentration levels in 1A and 1B groups.

#### 3.1.3. Differences in MMP-2 and MMP-3 Concentrations in the Corneal Epithelium

Since no differences were found in MMP-2 and MMP-3 concentrations between 1A and 1B groups, we decided to determine the levels of these two matrix metalloproteinases in group 1 (as a total with no subdivision into 1A and 1B) and group 2. Extremely high values of MMP-2 and MMP-3 were included in the initial analysis but excluded from further analyses. The mean MMP-2 and MMP-3 concentrations were higher in group 1. The same was noted after the exclusion of the extreme values.

An analysis of MMP-2 and MMP-3 revealed their concentrations were significantly higher in group 1 compared to group 2. The results are presented in Table 3.

#### 3.1.4. MMP-2 and MMP-3 Concentrations in the Corneal Epithelium by Sex

(1) MMP-2 concentration in the corneal epithelium by sex: Although MMP-2 concentrations were higher in males (621.1 ± 863.9) compared to female participants (504.4 ± 830.6), an analysis of the entire study population (groups 1 and 2) did not reveal statistically significant differences in MMP-2 levels between the sexes: *U* = (*N*_women_ = 61, *N*_men_ = 45) = 1145; *Z* = 1.45; *P*=0.1.

**Figure 4:**
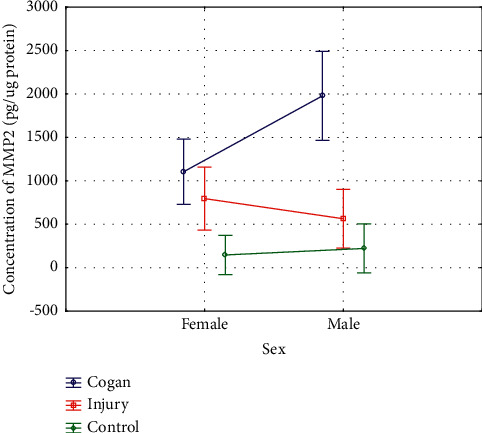
Assessment of the influence of sex and group on the values of MMP-2 concentration.

A two-way ANOVA with sex and group as factors affecting MMP-2 levels showed a statistically significant interaction between sex, group (1A, 1B, 2), and MMP-2 concentrations: *F* (2, 103) = 3.792, *P*=0.02 (Figure 4).

Generalized linear models did not reveal any interaction between sex, RCE duration, or age at corneal epithelium collection and MMP-2 concentrations.

(2) MMP-3 concentration in the corneal epithelium by sex: Although MMP-3 concentrations were higher in female (106.1 ± 183.5) compared to male participants (75.9 ± 98.4), an analysis of the entire study population (groups 1 and 2) did not reveal any statistically significant differences in MMP-3 levels between the sexes: *U* = (*N*_women_ = 51, *N*_men_ = 39) = 967; *Z* = −0.21; *P*=0.8.

**Figure 5:**
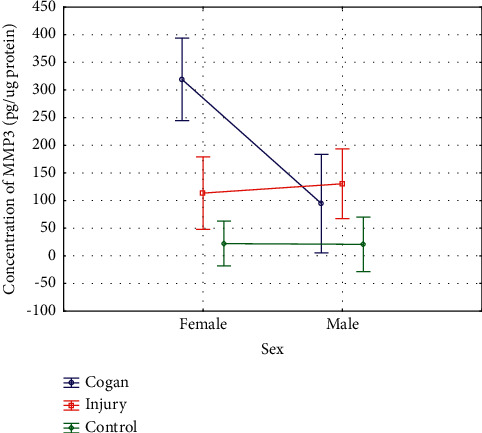
Assessment of the influence of sex and group on the values of MMP-3 concentration.

A two-way ANOVA with sex and group as factors affecting MMP-3 levels showed a statistically significant interaction between sex, group (1A, 1B, 2), and MMP-3 concentrations: *F* (2, 95) = 6.436, *P*=0.002 (Figure 5).

Generalized linear models did not reveal any interaction between sex, RCE duration, or age at corneal epithelium collection and MMP-3 concentrations.

#### 3.1.5. MMP-2 and MMP-3 Concentrations in Group 1 by Epithelial Stability after Phototherapeutic Keratectomy

(1) MMP-2 concentrations in group 1 by epithelial stability after phototherapeutic keratectomy: MMP-2 concentrations in patients with unstable and stable corneal epithelium (7 days of PTK) were 852.9 ± 1026.7 and 1020.72 ± 1122.2, respectively. The difference proved statistically nonsignificant: *U* = (*N*_instable epithelium_ = 16, *N*_stable epithelium_ = 34) = 241; *Z* = −0.63; *P*=0.5.

(2) MMP-3 concentrations in group 1 by epithelial stability after phototherapeutic keratectomy: MMP-3 concentrations in patients with unstable and stable corneal epithelium (7 days of PTK) were 245.2 ± 263.6 and 127.8 ± 146.3, respectively. The difference was statistically nonsignificant: *U* = (*N*_instable epithelium_ = 13, *N*_stable epithelium_ = 31) = 142; *Z* = −1.51; *P*=0.1.

#### 3.1.6. MMP-2 and MMP-3 Concentrations in Group 1 by Pre-PTK Symptom Duration

The Spearman rank correlation test did not reveal any correlation between MMP-2 and disease duration in the 1A group: *R* = 0.1; *P*=0.6. In the 1B group, however, the correlation strength was *R* = 0.01; *P*=0.9. An analysis of MMP-3 in group 1 (as a whole) did not show any correlation between MMP-3 and disease duration (*R* = 0.007; *P*=0.9). However, a moderate and positive correlation between these variables was revealed in 1A (*R* = 0.38; *P*=0.1) while in 1B the correlation was poor and negative (*R* = −0.21; *P*=0.2). Therefore, no relationship was found between disease duration and MMP-2 and MMP-3 concentrations.

## 4. Discussion

The studies carried out so far showed that MMP-2 was expressed in intact corneal epithelium and stroma as a proenzyme [29–32]. This is consistent with our observations; we confirmed the presence of MMP-2 in the healthy corneal epithelium of the control participants. The mean MMP-2 concentration in group 2 was 185.1 pg/*μ*g total protein ±117.1; the concentrations were higher in men. It is believed that MMP-2 might play a major regulatory role in the corneal stroma getting activated to catalyze the cleavage of damaged collagen fibrils [33]. MMP-2 was found to correlate with the period of keratocyte migration or activation at the injury site; it was also revealed that MMP-2 was present behind the leading edge of migrating epithelium which might indicate its role in long-term stroma remodeling and basement membrane resynthesis [28, 30, 34, 35]. Fini et al. assessed the role of MMP-2 in delayed corneal re-epithelialization after chemical burns in rats. They showed that failure to re-epithelialize correlated with increased amounts of gelatinolytic matrix metalloproteinases in the rat cornea. MMP-2 expression increased on day 1 postinjury at the site of fibroblast influx. It continued to rise for a few weeks; peak expression was proportional to the number of fibroblasts accumulated in the repaired tissue. These data are supportive of the concept that overexpression of matrix metalloproteinases by resident corneal cells interferes with re-epithelialization after some types of corneal injury [29]. It was also shown that MMP-2 was secreted not only by fibroblasts but also by immune cells migrating to the wound bed [36, 37].

Our RCE patients (Cogan's microcystic dystrophy and posttraumatic corneal erosions) had significantly higher MMP-2 levels than the control participants with the stable epithelial-stromal interface. These results are consistent with other studies on MMP-2 and MMP-9 activities in the corneal epithelium and the effect thereof on recurrent corneal erosions. MMP-2 expression was found to be increased in patients with RCEs compared to samples obtained from healthy individuals [21]. Measurements of gelatinolytic matrix metalloproteinases activity in tears seem to indicate that MMP presence in remission might be a predictor of recurrent disease [22].

MMP-3 analyses revealed low levels (26.6 pg/*μ*g total protein ±35.1) of this matrix metalloproteinase in healthy corneal epithelium. Li et al. also found mRNA MMP-3 expression in human corneal epithelial cells; MMP-3 production was stimulated by IL-1*β i* TNF-*α* [38].

This is the first study to evaluate MMP-3 levels and activity in patients with RCE. Hitherto published observations concerned MMP-3 activity after traumatic injuries or corneal refractive surgery. Weak and transient MMP-3 activity within corneal stroma has been revealed, correlating (but to a lesser extent than MMP-2 and MMP-9) with the period of keratocyte migration or activation at the wound site, basement membrane resynthesis, and stromal remodeling [28, 30, 31, 35, 36]. Similar to MMP-1 and MMP-2, MMP-3 expression in the stroma increases gradually following corneal injury. Its overexpression was noted at the sites of fibroblast accumulation and continued to rise for a few weeks. Peak expression was proportional to the number of fibroblasts accumulated in the repaired tissue [35].

In our study, MMP-3 values in the corneal epithelium of the RCE patients were significantly higher compared to the control. This is a novel finding, which might be of importance considering RCE pathogenesis. Within the study group, MMP-3 concentrations were higher in patients with Cogan's microcystic dystrophy (1A) than in those with posttraumatic RCEs (1B); however, the difference was not statistically significant. Hence, the results are comparable to MMP-2 although MMP-3 values are markedly lower.

We also evaluated those variables that might have some effect on MMP-2 and MMP-3 concentrations. No significant differential effects of male or female sex were found in the entire study population although men had higher MMP-2 levels while higher MMP-3 concentrations were noted in women. However, a two-way ANOVA revealed that, among patients with Cogan's microcystic dystrophy, MMP-2 was significantly higher in men and MMP-3 in women. The opposite was found for posttraumatic RCEs, i. e., MMP-2 was significantly higher in women and MMP-3 in men. No correlation was revealed between RCE duration and MMP-2 and MMP-3 concentrations. No significant correlations were also found between MMP-2 and MMP-3 elevation and post-PTK epithelial stability. These are important findings that should be the subject of further and comprehensive research.

Our analysis did not reveal any correlation between the age of the patients and MMP-2 concentration in the corneal epithelium in the entire study group or any of the subgroups. However, a moderate and positive correlation was found between MMP-3 levels and the age of the study group (group 1). However, the stratified analysis showed a loss of this correlation in groups 1A and 1B. It should be emphasized though that generalized linear models that were used to analyze the simultaneous effects of sex, age, or RCE duration on MMP-2 and MMP-3 did not show any interaction between these variables. Since no studies have reported similar results, we cannot provide any comments regarding this issue. However, it might be well-justified and interesting to combine MMP determinations in the corneal epithelium and the patient's blood sample. This might also allow the examiners to find out whether MMP levels change with age.

To our best knowledge, this is the first study to quantitatively assess MMP concentrations in the corneal epithelium of RCE patients undergoing PTK. This is also the first time that MMP-2 and MMP-3 of RCE patients were analyzed with respect to disease etiology, sex, age, and symptom duration and also whether MMP-2 and MMP-3 levels correlate with the stability of post-PTK epithelium. Since MMP-1, MMP-7, MMP-8, and MMP-9 concentrations were below the assays' sensitivity, it may imply that they are not involved in the process of subsequent erosion recurrences. This may also suggest that MMP-2 and MMP-3 are mainly responsible for the development of the disease and should be further explored in the future, as they may potentially constitute the main target of therapy.

In a long-term perspective, knowledge of MMPs concentrations in the corneal epithelium of RCE patients might substantially contribute to the optimization of treatment strategies. Current literature data show attempts to use oral tetracycline and topical steroids to prevent the recurrence of corneal erosion by the mechanism of MMP inhibition [20, 39]. It has been shown that tetracycline may inhibit MMP-9 and some authors recommend its use for the prevention and treatment of RCE. However, its administration in the acute phase of the disease, when the concentration of MMP-9 remains at a high level, is fully justified, based on the results obtained in this study, its use in prophylaxis, in the remission phase raises more doubts. It was also confirmed that dexamethasone inhibits collagen degradation by inhibiting IL-1*β* - an interleukin that increases the release of MMPs by keratocytes. Moreover, dexamethasone inhibited not only the synthesis of MMPs by these cells both at the protein and mRNA levels but also the activation of these enzymes. It did not directly inhibit the enzymatic activity of MMPs [40]. Interesting observations were also noted when assessing the effect of cysteine on the process of corneal regeneration in the mechanism of MMP inhibition. It has been shown that cysteine significantly accelerates epithelial healing after excimer photoablation [41]. Oral cysteine supplementation reduces mean corneal wound healing time in patients after laser excimer procedure [33, 42]. In connection with the obtained results, it seems reasonable to conduct more studies to assess the effect of cysteine on the frequency of subsequent erosion recurrences in patients with RCE.

The determination of tissue inhibitors of matrix metalloproteinases (TIMPs) is another important issue that encourages further studies as they could become new drug therapies. Hence, further research in this area is well warranted.

## 5. Conclusions

MMP-2 and MMP-3 concentrations were significantly higher in patients diagnosed with Cogan's microcystic dystrophy and posttraumatic RCEs compared to the control participants with the stable epithelial-stromal interface. The differences in concentrations of these two matrix metalloproteinases between patients with Cogan's microcystic dystrophy and those with posttraumatic RCEs were not statistically significant. The results can prove that MMP-2 and MMP-3 can dissolve anchoring fibers and the corneal epithelial basement membrane could be responsible for recurrent corneal erosions.

Despite the use of ultrasensitive assay kits, the obtained MMP-1, MMP-7, MMP-8, and MMP-9 concentrations were not adequate for analysis in the study and control groups. Due to the inability to assess the abovementioned MMPs in the tested material, it would be worth reassessing their concentration in the corneal epithelium. Particular attention should be paid to the MMP-1 and MMP-9 due to their substrate specificity and increased activity in the acute phase of corneal erosion. MMP-7 and MMP-8 may also be of potential importance due to their properties degrading the elements of the epithelial-stromal interface.

## Figures and Tables

**Table 1:** Patient characteristics.

Variable	Group			*P* value (1A vs. 1B)
	1A; *n* = 22	1B; *n* = 34	2; *n* = 65	
Sex W, *n*; % M, *n*; %	15; 20.8% 7; 14.3%	17; 23.6% 17; 34.7%	40; 55.6% 25; 51%	0.3

Age (years)	54.44 ± 15.0	36.2 ± 9.7	28 ± 5.6	<0.001
Age at RCE onset (years)	51.9 ± 14.8	33.3 ± 8.2	n/a	<0.001
Pre-PTK symptom duration (months)	24 (12–46)	19.5 (7–31)	n/a	0.3

Epithelial instability Yes, *n*; % No, *n*; %	9; 41% 13; 59%	9; 26% 25; 74%	n/a	0.2

Cause of injury Finger, *n*; % Other, *n*; %	n/a	14; 41% 20; 58%	n/a	n/a

n/a, not applicable; W, women; M, men; RCE, recurrent corneal erosions.

**Table 2:** MMP-2 and MMP-3 concentration values in the analyzed groups.

Variable		Group			*P* value
		1A, *n* = 22	1B, *n* = 34	2, *n* = 65	
MMP-3	$\bar{X}$ ; SD	226.5 ± 258.5	122.1 ± 127.0	26.6 ± 35.1	<0,0001
	Me; IQR	173.0 (4.4–21, 21–265)	68.9 (3.7–15, 15–196)	14.8 (7.8–27.6)	

MMP-2	$\bar{X}$ ; SD	1410.3 ± 1471.8	671.5 ± 586.4	185.1 ± 117.1	<0,0001
	Me; IQR	761.9 (183.0–1151.3)	566.3 (183.0–586.4)	167.0 (104.1–228.6)	

$\bar{X}$ ± SD, mean value ± standard deviation after removing the extreme values; Me ± IQR, median ± quartile range without removing extreme values.

**Table 3:** Values of MMP-2 and MMP-3 concentrations in groups 1 and 2 with and without outliers.

Variable	Statistics	Group 1A *i* 1B	Group 2	*P* value
MMP-2	$\bar{X}$ ±SD	967.0 ± 1084.9	175.7 ± 121.2	<0.0001
	Me ± IQR	596.0 (183.0–1325.0)	160.4 (96.6–228.0)	
	Min–max	56.2–4500.7	0–549.8	

MMP-3	$\bar{X}$ ±SD	281.7 ± 471.6	21.5 ± 33.2	<0.0001
	Me ± IQR	118.6 (18.6–302.9)	10.6 (2.86–23.5)	
	Min–max	2.0–2369.6	0–155.5	

MMP-2^*∗*^	$\bar{X}$ ±SD	967.0 ± 1084.9	185.1 ± 117.1	<0.0001
	Me ± IQR	596 (183.0–1325.3)	167.0 (104.1–228.6)	
	Min–max	56.2–4500.7	18.6–549.8	

MMP-3^*∗*^	$\bar{X}$ ±SD	162.5 ± 193.0	26.8 ± 35.1	<0.0001
	Me ± IQR	105.6 (16.2–238.4)	14.8 (7.8–27.6)	
	Min–max	2.0–926.0	1.6–155.5	

^*∗*^Analysis without outliers; $\bar{X}$ ± SD, mean value ± standard deviation after removing the extreme values; Me ± IQR, median ± quartile range without removing extreme values; Min–max, minimum–maximum values.

## Data Availability

The data supporting the findings of this study are included within the article.
